# Fibroblast Growth Factor 2-engaged cell spheroid for stem cell therapy: role of Interleukin 8 in the immune-modulatory effectiveness in the critical limb ischemia model

**DOI:** 10.1093/stcltm/szaf051

**Published:** 2025-11-14

**Authors:** Eunyeong Kim, Yunji Joo, Jong-Wan Kim, Sang-Heon Kim

**Affiliations:** Center for Biomaterials, Biomedical Research Institute, Korea Institute of Science and Technology (KIST), Seoul 02792, Republic of Korea; Division of Bio-Medical Science and Technology, KIST School, Korea University of Science and Technology, Seoul 02792, Republic of Korea; Center for Biomaterials, Biomedical Research Institute, Korea Institute of Science and Technology (KIST), Seoul 02792, Republic of Korea; Division of Bio-Medical Science and Technology, KIST School, Korea University of Science and Technology, Seoul 02792, Republic of Korea; Department of Research and Development, S.Biomedics Co., Ltd, Seoul 04797, Republic of Korea; Center for Biomaterials, Biomedical Research Institute, Korea Institute of Science and Technology (KIST), Seoul 02792, Republic of Korea; Division of Bio-Medical Science and Technology, KIST School, Korea University of Science and Technology, Seoul 02792, Republic of Korea

**Keywords:** CLI, FGF2, IL8, immune modulation, stem cell spheroids

## Abstract

**BACKGROUND:**

Stem cell therapy has emerged as a promising approach for treating critical limb ischemia (CLI), a condition caused by atherosclerosis that results in reduced blood flow and limb necrosis. However, the underlying therapeutic mechanisms involving factors secreted from stem cells are still in the early stages of exploration. This study focuses on investigating the tissue regenerative effects of interleukin-8 (IL8) secreted from cell spheroids.

**METHODS:**

Human adipose-derived stem cells (hASCs) were cultured on FGF2-tethered surfaces to form spheroid (FECS-Ad). A murine CLI model was established through femoral artery dissection, followed by the injection of various treatments, including PBS, hASC, FECS-Ad, IL8-silenced FECS-Ad, and recombinant IL8.

**RESULTS:**

Comparative analyses revealed that FECS-Ad injection resulted in a higher percentage of salvaged limbs, but these effects were attenuated when IL8 was silenced in FECS-Ad. Immunofluorescence staining, flow cytometry analysis and RT-qPCR of M1 and M2 macrophage markers demonstrated that IL8 has the ability to polarize macrophages to M2 type. Notably, FECS-Ad injection reduced apoptotic markers (caspase 8 and TUNEL) in ischemic tissues, whereas IL8 knockdown in FECS-Ad increased the proportion of apoptotic cells. FECS-Ad injected tissues showed larger regenerating muscle fibers with centrally located nuclei. Knockdown of IL8 in FECS-Ad decreased the area and size of regenerating muscle fibers.

**CONCLUSIONS:**

Our findings underscore the dual role of IL8 in safeguarding muscle tissues from degeneration and orchestrating immunomodulatory effects by finely tuning tissue inflammation and macrophage polarization. This study highlights IL8 as a pivotal paracrine factor contributing to tissue regeneration in the context of stem cell therapy for CLI.

## Graphical abstract

**Graphical Abstract:**
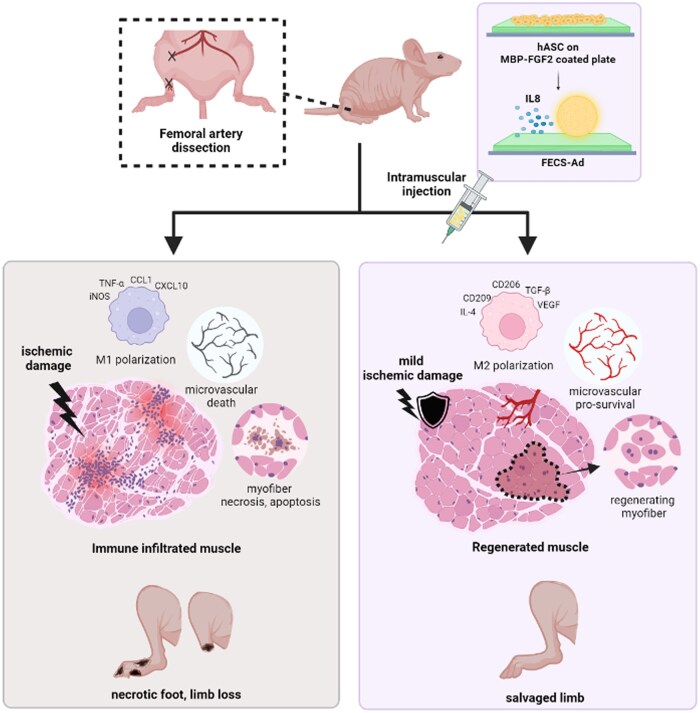

Significance statement The injection of human adipose-derived stem cells, cultured on FGF2-tethered surfaces to form spheroids, demonstrated healing effects by preventing limb necrosis and loss. Further analysis revealed that these effects are mediated through immune modulation, driven by interleukin-8 (IL8) secreted from the stem cell spheroids. This study clarifies the previously unclear mechanisms underlying stem cell therapy for critical limb ischemia, particularly the role of factors secreted by stem cells. While IL8 was historically thought to solely promote inflammation and contribute to chronic inflammation, our findings show that, when administered at the appropriate concentration, IL8 can modulate inflammation and facilitate tissue regeneration in critical limb ischemia. These results highlight IL8’s potential as a key factor in future stem cell therapies. The use of stem cell spheroids resulted in a sustained healing effect, altering the injury environment dynamics.

## Introduction

Critical limb ischemia (CLI) is a severe manifestation of peripheral artery disease (PAD) caused by atherosclerosis, resulting in reduced blood flow and necrosis to limbs.^1^ Patients with CLI face a high risk of amputation or cardiovascular death. Although surgical interventions such as angioplasty and bypass surgery are commonly employed to address CLI, they often prove unsuccessful due to limitation in revascularization among CLI patients.^2^ Stem cell therapy is gaining traction as a prominent treatment modality for CLI, with documented efficacy.^3^ Nevertheless, investigations into the therapeutic mechanism underlying the secretion of factors from stem cells are at a nascent state of exploration.

Adipose-derived stem cells (ASC) belong to the category of mesenchymal stem cells, offering regenerative therapeutic effects by differentiating into various tissue progenitors, migrating to damaged regions, and leveraging autocrine and paracrine mechanisms for repair.^4^ Human ASC (hASC) are frequently utilized in treating CLI patients due to their ability to promote new capillary formation by secreting angiogenic factors like vascular endothelial growth factor (VEGF), fibroblast growth factor 2 (FGF2), and platelet-derived growth factor (PDGF). Moreover, hASC are easily obtained via liposuction, a less invasive way compared to obtaining bone marrow-derived stem cells.^5^^,^^6^ However, when transplanted in vivo, individual hASC exhibit low survival and retention rates, thereby limiting therapeutic efficiency.^7^ To address these limitations, we previously developed hASC spheroids that secrete various angiogenic factors, including interleukin-8 (IL8).^8^ To form a spheroid, hASC were cultured on a hydrophobic polystyrene surface with immobilized maltose-binding protein fused with fibroblast growth factor 2 (MBP-FGF2). This setup facilitated weak cell adhesion between hASC and MBP-FGF2, allowing aggregation and self-organization into a spherical microtissue, namely FGF2-engaged cell spheroid of hASC (FECS-Ad).^9^ Compared to conventional monolayered hASC, FECS-Ad demonstrates improved retention and survival rates when transplanted in vivo, leading to enhanced therapeutic efficiency in mouse CLI models by providing increased paracrine and angiogenic factors.^8–10^

Interleukin-8 is a key paracrine and angiogenic factors secreted by FECS-Ad.^8^^,^^9^ Recent evidence suggests that an MBP-FGF2 presented surface primes hASC through the FGF2-FGF receptor 1 (FGFR1) signaling cascade. The FGF2-FGFR1 signaling of hASC on the MBP-FGF2 matrix triggers the nuclear factor kappa-light-chain-enhancer of activated B cells (NF-κB) signaling cascade, leading to increased IL8 secretion, subsequent VEGF, and enhanced angiogenesis.^8^ IL8 is known primarily for its role in angiogenesis and endothelial cell proliferation, survival, and migration, as well as in the recruitment of neutrophils and monocytes.^11^^,^^12^ IL8 is recognized as a pro-inflammatory cytokine involved in the recruitment and activation of neutrophils, which are key players in the acute inflammatory response.^13^ However, recent research has indeed shown that IL8 can also play a role in the polarization of macrophage into anti-inflammatory M2 phenotype.^14–17^

Macrophages play crucial roles in tissue regeneration by transitioning between phagocytic pro-inflammatory M1 phenotype and regenerative anti-inflammatory M2 phenotype throughout the regeneration process.^18^ Macrophages in ischemia-damaged tissues in amputated limbs from CLI patients predominantly comprise pro-inflammatory macrophages expressing TNF and IL1β.^13^ Furthermore, the pro-inflammatory niche of the ischemic limbs is related to the premature differentiation of muscle stem cells, resulting in the muscle regeneration failure and considerable tissue loss.^13^ Therefore, anti-inflammatory macrophages are essential to prevent the worsening of the inflammatory response in CLI patients and to facilitate muscle regeneration. In addition to averting pro-inflammatory response, it is imperative to promptly prevent or mitigate tissue degeneration, necrosis, and apoptosis.^19^ Several studies suggest that IL8 promotes cell proliferation and inhibits apoptosis in prostate cancer and human hepatocytes.^20^^,^^21^

Successful tissue regeneration requires the distribution of necessary nutrients and components through revascularized vessels and modulation of the immune response during the early stages.^22^ Our previous research has elucidated how the increased IL8 from FECS-Ad positively influences angiogenesis and overall tissue regeneration in mouse CLI model. In this study, we explored whether IL8 from FECS-Ad participates in 2 other crucial aspects of regeneration: immune modulation and tissue protection in a CLI model. Based on the M2 polarization effects of IL8, we aimed to investigate its potential in activating regenerative M2 macrophages and inducing an anti-inflammatory microenvironment in a mouse CLI model, thereby promoting muscle regeneration. In addition, we examined the anti-apoptotic effects of IL8 derived from FECS-Ad in the ischemia-damaged muscle. The findings of our study offer novel insights into its role in treating ischemic injuries.

## Methods

### hASC culture and formation of FECS-Ad

Human adipose-derived stem cells were obtained from S.Biomedics and cultured up to the fifth passage for experimental use in human MSC growth medium (CEFO bio; CEFOgro-MSC) according to the manufacturer’s instructions. The medium was changed every other day, and cells were subcultured using 0.25% trypsin-EDTA (Corning; 25-053-CI). For the formation of FGF2-engaged cell spheroid of hASC (FECS-Ad), recombinant MBP-FGF2 fusion protein was produced in *E coli* following the method outlined previously.^10^^,^^23^ Briefly, the pMAL-c2X vector (New England Biolabs), containing the human FGF2 cDNA (Bioneer), was constructed and introduced into *E coli* strain BL21(DE3) (New England Biolabs). The MBP-FGF2 protein expressed in *E coli* was purified according to the manufacturer’s protocol (New England Biolabs). For the experiment, MBP-FGF2 (at 20 µg/mL in phosphate-buffered saline [PBS]) was added to each well (50 µL per well) of 384-well clear polystyrene microplates (Corning) and incubated for 4 h at 23 °C. After coating the plates, MBP-FGF2 solution was removed from each well by washing 3 times with PBS, and hASC, detached by trypsinization, were suspended in StemPro MSC serum-free medium (Gibco; A1067501) and seeded at a concentration of 1 × 10^4^ cells per well. The seeded cells were incubated for 24 h in the presence of FGF2 in a 37 °C incubator with 5% CO_2_. Complete spheroid formation was observed under a phase contrast microscope (Carl Zeiss; AxioVert A1) and photographed using a digital optical camera (Pentax; Pentax K-1) with a Tamron 90 mm F/2.8 macro lens (Tamron) (Figure S1A, see online supplementary material for a color version of this figure).

### siRNA transfection for preparation of IL8 knocked-down FECS-Ad

To knock down IL8 expression in hASC, the cells were transfected with a final concentration of 10 μM IL8 siRNA (Santa Cruz Biotechnology; sc-37007) using Lipofectamine RNAiMAX (ThermoFisher Scienfic; 13778030) as the delivery vehicle, following the manufacturer’s instructions. Briefly, for every 1 × 10^6^ cells, 9 μL of Lipofectamine RNAiMAX Reagent was diluted in 150 μL of Opti-MEM (Gibco, 31985-070), while the siRNA was diluted in a separate 150 µL of Opti-MEM. The siRNA solution was then added to the Lipofectamine solution and incubated for 5 min. The combined solution was applied to hASC that had reached 60%-80% confluency. IL8 knockdown (IL8 KD) was confirmed using RT-qPCR and ELISA, and control siRNA was used to assess and prove off-target effects (Figure S1B and C, see online supplementary material for a color version of this figure).

### Animal care

Animal handling was conducted in accordance with the guidelines set by the National Institutes of Health of South Korea. Six-week-old male BALB/c nude mice were purchased from Orient Bio Inc. for in vivo studies. All mice had ad libitum access to food and water and were allowed to acclimatize for at least 1 week prior to experimentation. During the study, the mice were housed in an environment maintained at 24 °C with 60% humidity with a 12-h light-dark cycle. All experiments were approved in compliance with the animal care guideline set by the International Animal Care and the Institutional Animal Care and Use Committee of the Korea Institute of Science and Technology [KIST-2022-145].

### Hindlimb ischemia induction and FECS-Ad administration

Twenty-four six-week-old male BALB/c nu/nu mice were anesthetized using 3% isoflurane inhalation in 100% oxygen and subjected to unilateral hindlimb ischemia (HLI) induction. The right leg was opened, and the femoral artery was excised from the proximal point of the external iliac artery to the distal bifurcation into the saphenous and popliteal arteries. The indicated points were tied off with 5-0 silk suture (AILEE; SK526), and the artery was completely removed. Blood flow blockage was monitored using Laser Doppler imaging (LDI; Moor Instruments; moorLDI2-HIR). The mice were randomly divided into 4 groups (*n* = 6 per group) and given 4 different treatments 1 day after surgery: (1) PBS as a negative control, (2) hASC, (3) FECS-Ad, and (4) IL8 KD FECS-Ad. A total of 5 × 10^5^ cells suspended in 0.2 mL of PBS (equivalent to 50 spheroid cells) were administered to the adductor, gracilis, and pectineal muscles, with equal amounts for each injection in the medial thigh of the ischemic limb, as previously described.^10^ The extent of necrosis was assessed using the following grading scale: grade 0, limb salvage; grade 1, necrosis of some toes; grade 2, whole toe necrosis; grade 3, necrosis extending to foot; and grade 4, limb loss. The medial thigh tissue where the cell administrations were made was harvested at 3 and 7 days post-HLI induction for further experiments.

For the injection of recombinant human IL8 (rhIL8) in the CLI model, rhIL8 (R&D systems; 208-IL) was dissolved in PBS at concentrations of 500, 1000, and 1500 ng/mL. Each concentration was prepared in a total volume of 0.2 mL PBS, corresponding to the rhIL8 1×, 2×, and 3× groups, respectively (*n* = 6 per group). The rhIL8 solution was administered 1 day after HLI surgery. Medial thigh tissue was harvested at 3 and 7 days post-surgery for immunofluorescence staining.

### Immunofluorescence staining and histological analysis

Thigh muscles were harvested on days 3 and 7, then prepared as paraffin blocks and sectioned into 6 μm-thick slices. All sections were deparaffinized, washed twice with PBS, and blocked with 2% BSA (Bovogen; BSAS0.1) and 0.1% Triton X-100 (Sigma; T8787) in PBS for 1 h. Samples were incubated with primary antibodies diluted in blocking buffer overnight at 4 °C. Following 3 washes in PBS, sections were incubated with Alexa Fluor-conjugated secondary antibodies (Invitrogen) in blocking buffer for 1 h at room temperature. Samples were then mounted with Vectashield antifade mounting medium containing DAPI (Vector Laboratories Inc.; H1800) and examined using a confocal microscope (Zeiss; Zeiss LSM 700). Fluorescence images were analyzed using ImageJ software (National Institutes of Health). Information on antibodies is provided in supplementary files.

For histological analysis, thigh muscles were fixed in 4% paraformaldehyde and embedded into paraffin blocks. Paraffin sections with 6 μm thickness were deparaffinized, rehydrated, and stained with hematoxylin and eosin (H&E) according to standard protocols. Images were captured using a phase contrast microscope (Zeiss; Vert A1).

### Flow cytometry analysis

At the specified time points of 1, 3, and 7 days post-surgery, mice were euthanized using CO_2_, and ischemic muscle tissues were harvested. To obtain a single-cell suspension, the collected muscle tissue was digested in a solution containing collagenase (600 U/mL) and hyaluronidase (200 U/mL) (STEMCELL Technologies; 07912). The resulting cell suspension was then filtered through 70-μm and 40-μm cell strainers (Corning; 431750, 431751). The isolated cells were blocked with an anti-mouse CD16/32 antibody (BioLegend; 101302) and subsequently incubated for 1 h at 4 °C with fluorescently conjugated antibodies targeting the following mouse antigens: APC anti-F4/80 (pan-macrophage), PE anti-CD206 (M2 macrophage), and FITC anti-CD86 (M1 macrophage) (BioLegend; 123116, 141706, 105006). Flow cytometric analysis was performed using a Beckman Coulter instrument, with gates for separating negative and positive cells determined using isotype antibody control probes.

### Statistical analysis

All data are expressed as mean ± SD or ± SEM from 3 or more independent experiments. One-way ANOVA by Tukey’s test for multiple comparisons was used for statistical significance determination. All graphs and statistical significance were generated using GraphPad Prism 7 software (GraphPad). Technical and/or biological replicates and the number of experimental conditions are indicated in the figure legends.

## Results

### IL8 from FECS-Ad protects from tissue degeneration in murine model of hindlimb ischemia

**Figure 1.**
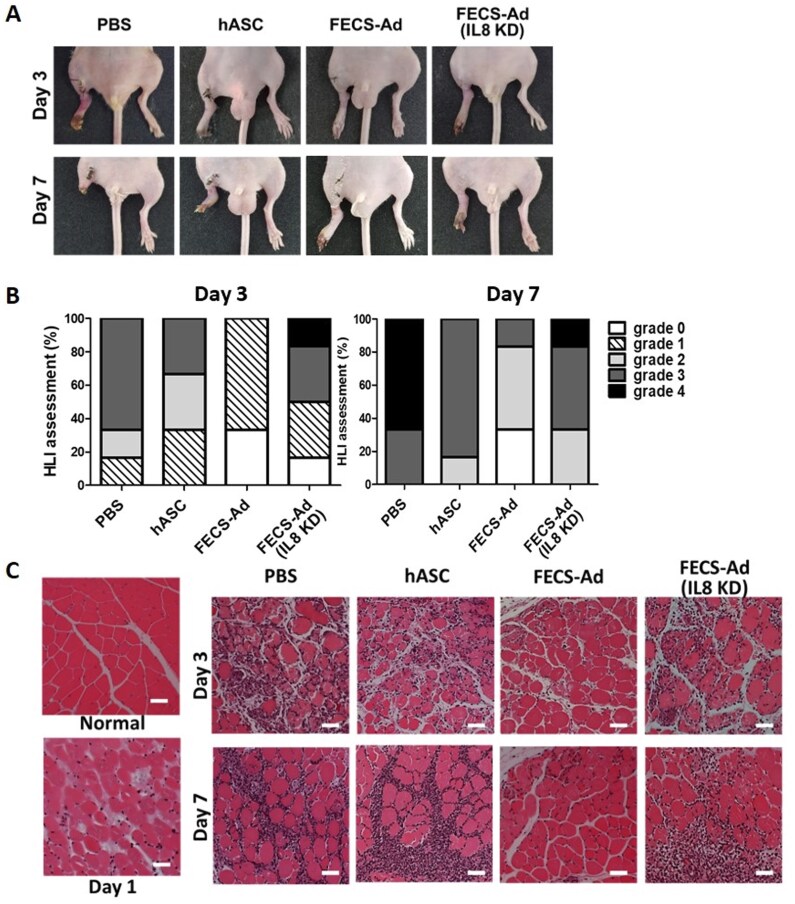
Protective potential of IL8 (interleukin-8) secreted from FECS-Ad (FGF2-engaged cell spheroid of hASC) in a hindlimb ischemia mouse model. (A) Representative images of hindlimb morphology following hindlimb ischemia (HLI) induction at days 3 and 7. Ischemia was induced in the limb, and one day after the surgery, mice were treated with phosphate-buffered saline (PBS), human adipose-derived stem cells (hASC) suspension, FECS-Ad, or IL8 knocked-down (IL8 KD) FECS-Ad. *n* = 6 per group. (B) Analysis of the physiological status of the ischemic limb assessed on days 3 and 7, as described (grade 0, limb salvage; grade 1, necrosis of some toes; grade 2, whole toe necrosis; grade 3, necrosis extending to foot; and grade 4, limb loss). HLI assessment presented the number of animals in each grade as a percentage. *n* = 6 per group. (C) Histological images of ischemic thigh muscle stained with hematoxylin and eosin on days 3 and 7. Scale bar = 50 μm.

To determine the role of IL8 in the regenerative phenomenon that occurs early after transplanting stem cells into ischemic hindlimbs of murine model, the pathological characteristics of the damaged tissue area were observed 3 and 7 days after stem cell transplantation. Evidence of damage was observed with the presence of necrotic toes and foot (Figure 1A). The extent of necrotic damage was evaluated by a detailed injury assessment using ischemia scoring as described in method (Figure 1B). In the mice injected with PBS and hASC, damage had extended to all toes and the entire foot by day 7, leading to limb loss in the PBS-injected group. FECS-Ad-injected group exhibited the highest percentage of salvaged limbs with damage limited to the toes. Conversely, IL8 gene knockdown in FECS-Ad resulted in severe damage that progressed to the entire foot (Figure 1A and B).

Histological differences were evaluated by performing H&E staining on cross-sections from the thigh muscle adjacent to the severely affected feet and toes. On day 3, a significant number of nuclei in the interstitial space was noted, suggesting the infiltration of immune cells necessary for the removal of impaired muscle fibers in groups injected with PBS and hASC (Figure 1C). This was accompanied by necrotic muscle fibers, as evidenced by the loss of fibrous integrity and the presence of invading mononuclei in the cytoplasm. On day 7, immune infiltration progressed to the endomysium and perimysium of the tissues injected with PBS, hASC, and IL8-silenced FECS-Ad. In contrast, immune infiltration and necrosis in the FECS-Ad injected group were limited to some muscle fibers (Figure 1C).

At days 3 and 7 post-surgery, the limbs injected with FECS-Ad were retained better, both in terms of histology and overall condition, compared to the other injection groups. Since the effect of preventing immune infiltration and muscle degeneration was reduced in the IL8-silenced FECS-Ad injected group compared to the FECS-Ad injected group, it is suggested that IL8 may play a role in preventing immune infiltration and muscle degeneration after ischemia in the hindlimb.

### IL8 from FECS-Ad affects macrophage polarization toward anti-inflammatory phenotype and prevents worsening of inflammatory response in murine CLI model

**Figure 2.**
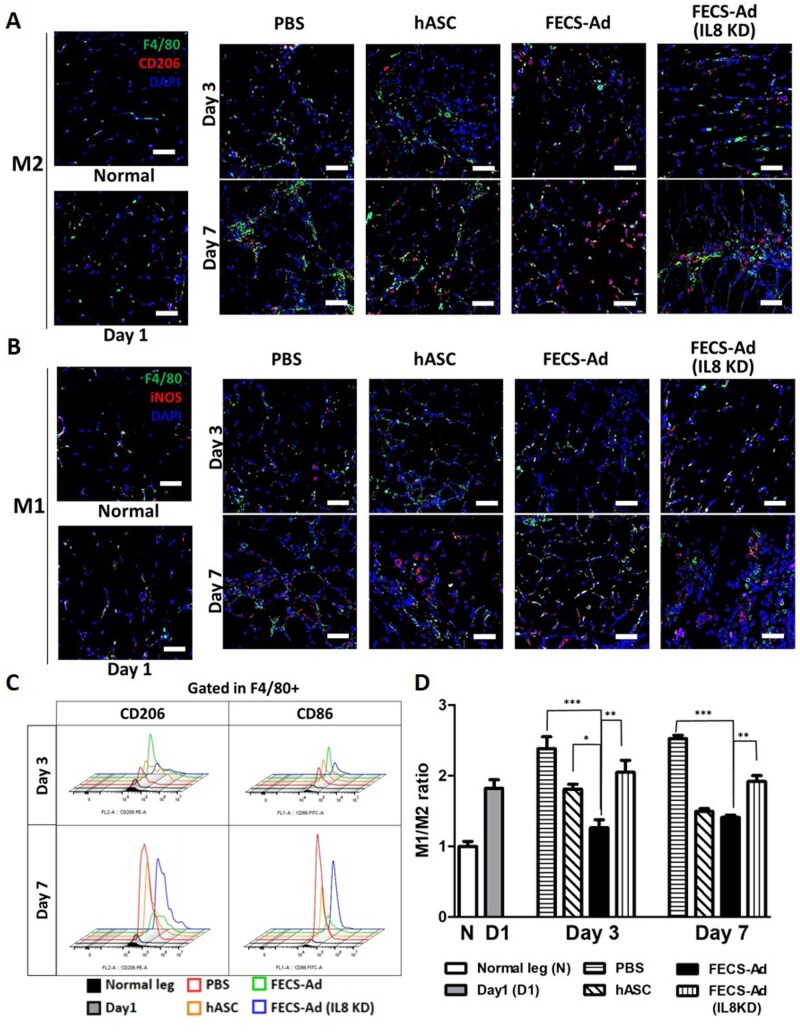
Anti-inflammatory modulation of interleukin-8 (IL8) from FGF2-engaged cell spheroid of hASC (FECS-Ad) in an ischemia-induced mouse hindlimb. (A, B) Ischemic thighs isolated from mice treated with phosphate-buffered saline (PBS), human adipose-derived stem cells (hASC), FECS-Ad, or IL8-silenced FECS-Ad were immunostained with F4/80 for pan-macrophages, CD206 for M2 macrophages, and iNOS for M1 macrophages at 1, 3, and 7 days post-surgery. Nuclei were stained with DAPI. *n* = 5 per group. Scale bar = 50 μm. (C) Representative histograms of flow cytometry analysis of mouse ischemic thighs. CD206-PE was used for gating M2 macrophages, and CD86-FITC was used for gating M1 macrophages within the F4/80-positive population. Isotype antibodies were served as negative controls. *n* = 3 per group (D) Quantification of the M1/M2 ratio based on cell number from flow cytometry. Corresponding values for panels (C) and (D) are provided in Table S1. Data are presented as mean ± SEM. ^*^P < .05, ^**^P < .01, ^***^P < .001, one-way ANOVA followed by Tukey’s test.

Macrophages have the ability to polarize into 2 distinct phenotypes, “M1” and “M2,” depending on the inflammatory environment.^24^^,^^25^ In order to investigate the effect of hASC, FECS-Ad, and IL8-silenced FECS-Ad injection on macrophage polarization at days 3 and 7 post-HLI induction, immunofluorescence staining of M1 and M2 macrophage markers was carried out (Figure 2A and B). Prior to cell injection, tissues were stained at day 1 post-HLI induction to observe the pathological state and phenotypes of recruited macrophages. F4/80 staining was used for pan-macrophage detection, iNOS for M1 macrophage identification, and CD206 for M2 macrophage identification. On day 1 after ischemic induction, activated macrophages expressing either iNOS or CD206 were observed in muscle tissues. By day 3, the proportion of M2 (CD206) among total macrophages (F4/80) increased in all groups, with the FECS-Ad injected group showing the highest increase. Interestingly, IL8 knockdown in FECS-Ad led to a decrease in M2 macrophages compared to those in the FECS-Ad injected group (Figure S2A, see online supplementary material for a color version of this figure). By day 7, the M2 macrophage ratio further increased in all groups, with the FECS-Ad injected group demonstrating the highest proportion (Figure S2B, see online supplementary material for a color version of this figure). The FECS-Ad injected group also exhibited lower M1 macrophages (iNOS) ratios on both day 3 and day 7, while IL8 knockdown in FECS-Ad resulted in an upregulation of M1 macrophages (Figure S2C and D, see online supplementary material for a color version of this figure). These results indicate that IL8 secreted from FECS-Ad affects the polarization of macrophage in the murine limb following ischemia induction, increasing the proportion of M2 macrophages and reducing the proportion of M1 macrophages.

We confirmed the modulation of macrophages toward an anti-inflammatory phenotype via IL8 secreted from FECS-Ad using flow cytometry. M1 macrophages were identified as being positive for F4/80 and CD86, while M2 macrophages were double positive for F4/80 and CD206. By day 3, M2 macrophages increased remarkably in FECS-Ad-injected tissues compared to the other groups. By Day 7, both M1 and M2 macrophages had rapidly increased in the PBS- and hASC-injection groups, indicating an exacerbating inflammatory response. In contrast, the macrophages in the FECS-Ad-injection group remained at levels similar to day 3, preventing the inflammatory response from aggravating. However, when IL8-silenced FECS-Ad was injected, the effect of polarizing toward anti-inflammatory macrophages disappeared by day 3, and an intense inflammatory response was observed on day 7 (Figure 2C). The M1/M2 ratio was obtained based on the number and proportion of cells obtained in flow cytometry analysis. The M1/M2 ratio increased on the first day of HLI, leading to the pro-inflammatory reaction. On day 3, the PBS- and hASC-injected groups had ratios that were higher or similar to those on day 1, while the FECS-Ad-injected group had a significantly lower ratio. This effect was maintained up to the seventh day. However, the FECS-Ad injection group with suppressed IL8 expression showed a similar ratio to day 1 on both days 3 and 7, confirming that anti-inflammatory modulation did not arise (Figure 2D). Additionally, gene expression analysis revealed that only the FECS-Ad injection upregulated anti-inflammatory markers on day 3 and prevented the increase of both pro- and anti-inflammatory markers by day 7, consistent with the findings in Figure 2C (Figure S3, see online supplementary material for a color version of this figure). We also confirmed the immune-modulatory effects of FECS-Ad in vitro through co-culture experiments with human macrophage and FECS-Ad (Figure S4, see online supplementary material for a color version of this figure). Taken together, the results suggest that IL8 secreted by FECS-Ad plays a significant role in modulating macrophage polarization in murine limb ischemia models.

### IL8 from FECS-Ad has influence over apoptosis in murine model of hindlimb ischemia

**Figure 3.**
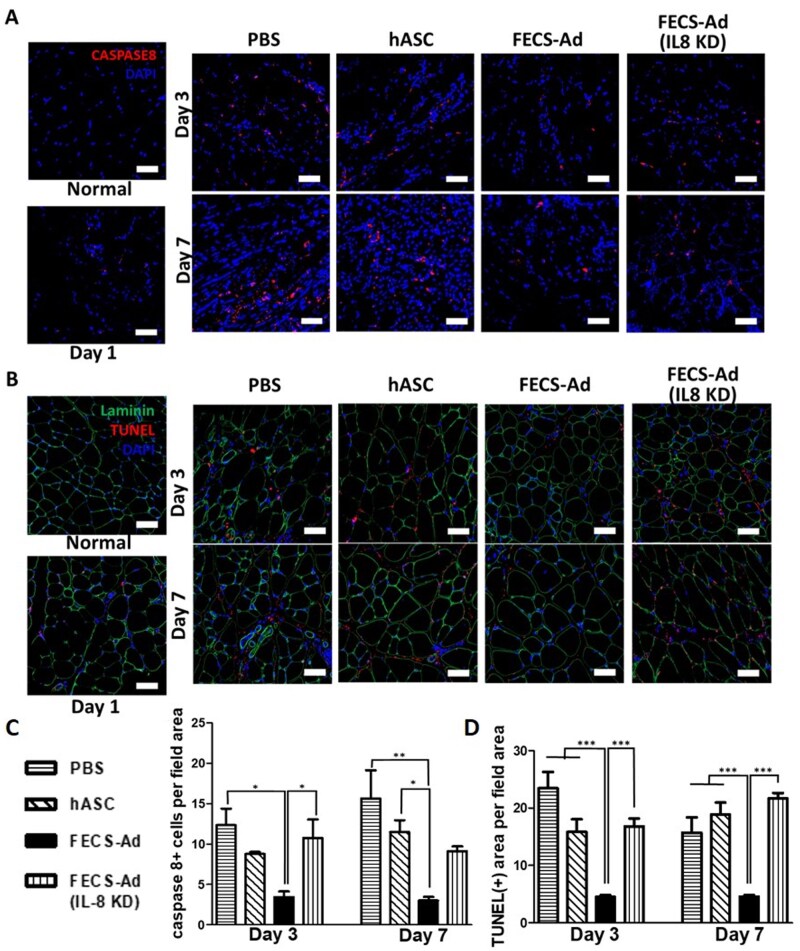
Roles of interleukin-8 (IL8) in the prevention of apoptosis by FGF2-engaged cell spheroid of hASC (FECS-Ad) in a mouse hindlimb ischemia. Immunofluorescent images showing apoptosis markers, caspase-8 (A) and TUNEL staining (B) in mouse medial thigh tissues at days 3 and 7 post-hindlimb ischemia (HLI) and treatment with phosphate-buffered saline (PBS), human adipose-derived stem cells (hASC), FECS-Ad, or IL8-silenced FECS-Ad. Nuclei were counterstained with DAPI. Scale bar = 50 μm. Quantification of caspase 8-positive cells (C) and TUNEL-positive area relative to field area (D). *n* = 5 per group. Data are presented as mean ± SEM. One-way ANOVA with Tukey’s test. ^*^P < .05, ^**^P < .01, ^***^P < .001.

To evaluate the effects of IL8 from FECS-Ad on tissue apoptosis following HLI, tissues were obtained at day 3 and day 7 from mice injected with PBS, hASC, FECS-Ad, and IL8-silenced FECS-Ad. Immunofluorescence staining for caspase 8, the initiator caspase of apoptosis, and TUNEL staining, used to detect DNA breaks during the final phase of apoptosis, were conducted (Figure 3A and B). Tissues collected at day 1, prior to cell injection, were examined to observe the effects of ischemia induction on apoptosis. At day 1, there were low levels of caspase 8 and TUNEL expressions. However, at days 3 and 7 after the ischemic induction, both expressions were significantly lower in the FECS-Ad-injected group compared to the PBS and hASC injected groups (Figure 3C and D). In the PBS- and hASC-injected groups, fragmented DNA was dispersed throughout the tissues, and the proportion of TUNEL-positive area was approximately 20% or higher. In contrast, the TUNEL-positive area in FECS-Ad injected tissues was about 5%. However, when IL8 was knocked down in FECS-Ad, the TUNEL-positive area increased to approximately 20% in the ischemic tissues (Figure 3B and D). These results indicate that FECS-Ad inhibits apoptosis in murine HLI, and IL8 may play a role in influencing apoptosis.

### IL8 from FECS-Ad promotes muscle regeneration in injured hindlimb by ischemia in murine model

**Figure 4.**
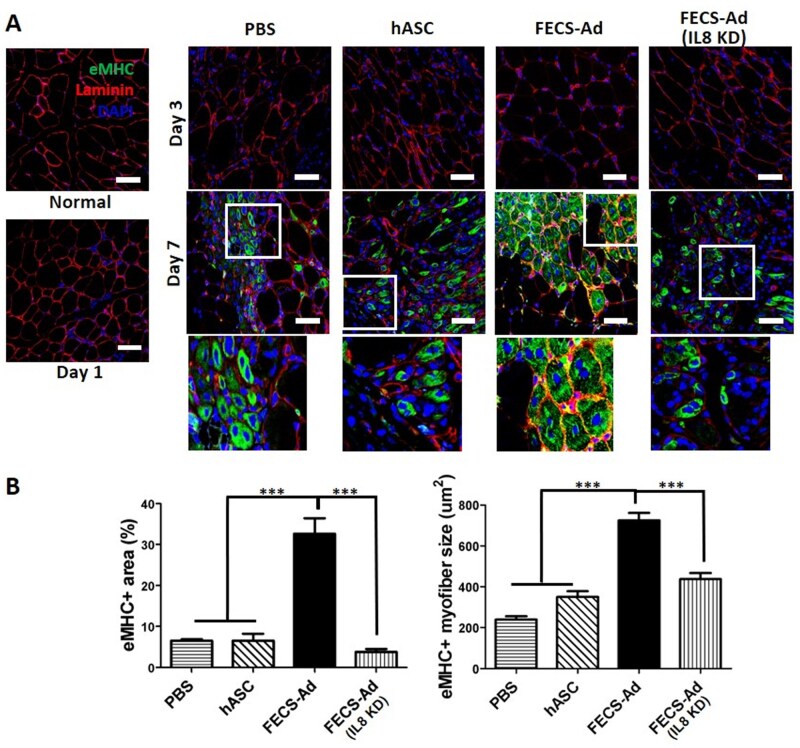
Effect of FGF2-engaged cell spheroid of hASC (FECS-Ad) on muscle regeneration in ischemic thigh muscle. (A) Immunofluorescent images of the medial thigh stained for laminin and embryonic myosin heavy chain (eMHC) following hindlimb ischemia (HLI) induction and treatment with phosphate-buffered saline (PBS), human adipose-derived stem cells (hASC), and FECS-Ad, interleukin-8 (IL8)-silenced FECS-Ad at days 3 and 7. *n* = 5 per group. Nuclei were counterstained with DAPI. Scale bar = 50 μm. (B) Quantification of eMHC area relative to total area and eMHC-positive myofiber size. Data are presented as mean ± SEM. *n* = 5 per group. One-way ANOVA with Tukey’s test. ^***^P < .001.

To investigate the effects of IL8 from FECS-Ad on muscle regeneration in hindlimb tissue after femoral artery dissection, we utilized embryonic myosin heavy chain (eMHC), which is expressed in developing muscle and during early stages of muscle regeneration, as well as laminin antibodies. On day 7, we observed eMHC-positive muscle fibers with centrally located nuclei in all groups, indicating muscle regeneration occurred following HLI. While the cross-net shape of muscle fibers, as indicated by laminin, was maintained in all groups, only a portion of the fibers with small and circular eMHC expression attempted to recover from the injury in the PBS and hASC injected tissues. The regenerating muscle fibers in the FECS-Ad injected tissues exhibited angular characteristics similar to those of normal tissue. However, the knockdown of IL8 resulted in smaller and circular morphologies in regenerating muscle fibers (Figure 4A). The tissues injected with PBS and hASC showed an eMHC-positive area of less than 10% of the field area. In contrast, the tissues injected with FECS-Ad exhibited an eMHC-positive area more than 3 times higher than that of PBS- and hASC-injected tissues (Figure 4B). In addition, the FECS-Ad injected group displayed the largest size of muscle fibers stained with eMHC (239.0 vs 350.6 vs 723.5 vs 437.3 μm^2^; *P* < .001; for PBS, hASC, FECS-Ad, and IL8-silenced FECS-Ad injected tissues, respectively). Conversely, IL8 knockdown in FECS-Ad resulted in a decrease in the eMHC-positive area and the size of regenerating muscle fibers in murine limb post-ischemia induction (Figure 4B). Overall, these results suggest that IL8 plays an important role in promoting muscle regeneration by FECS-Ad in ischemic muscle tissue.

### Recombinant human IL8 alone has anti-inflammatory and therapeutic effects in murine model of hindlimb ischemia

**Figure 5.**
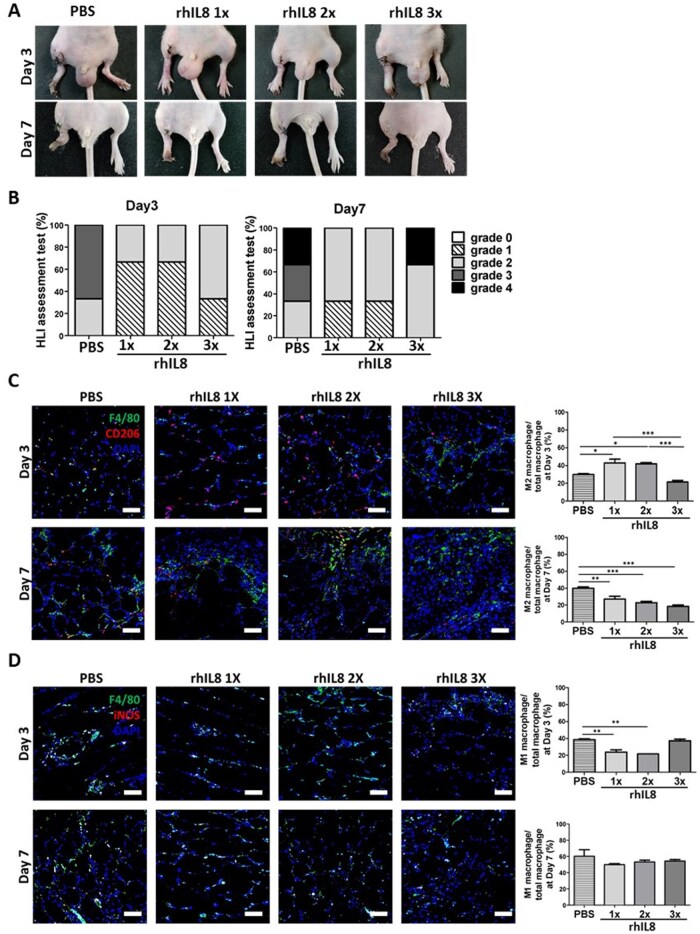
Effects of recombinant human interleukin-8 (rhIL8) in a murine model of hindlimb ischemia. (A) Representative images of hindlimb morphology following hindlimb ischemia (HLI) induction at days 3 and 7. Mice were treated with PBS, rhIL8 1×, 2×, and 3× doses on the first day post-surgery. *n* = 6 per group. (B) The extent of ischemic damage was scored on days 3 and 7, with grades defined as follows (grade 0, limb salvage; grade 1, necrosis of some toes; grade 2, whole toe necrosis; grade 3, necrosis extending to foot; and grade 4, limb loss). *n* = 6 per group. (C, D) Immunofluorescent images of the medial thigh stained for F4/80, CD206, and iNOS along with quantification of M2 or M1 macrophages as a proportion of total macrophages following HLI induction at days 3 and 7. *n* = 5 per group. Nuclei were counterstained with DAPI. Scale bar = 50 μm. Data are presented as mean ± SEM. *n* = 5 per group. One-way ANOVA with Turkey test. ^*^P < .05, ^**^P < .01, ^***^P < .001.

To investigate whether IL8 alone has anti-inflammatory and therapeutic effects, rhIL8 was applied to the CLI model. Based on the amount of IL8 secreted during the formation of FECS-Ad (Figure S1B, see online supplementary material for a color version of this figure), 1×, 2×, and 3× doses were injected on the first day of HLI induction. The degree of necrotic damage in hindlimb injected with rhIL8 1× and 2× was lower compared to the PBS group, and foot necrosis was prevented without loss of the lower extremities (Figure 5A and B). The proportion of CD206-positive macrophages in the rhIL8 1× and 2× injected groups significantly increased compared to the PBS group on day 3 post-surgery, but decreased on day 7 and was lower than that of the PBS-injected group, as observed by immunofluorescence staining (Figure 5C). The percentage of iNOS-positive macrophages in the rhIL8 1× and 2× injected tissues was also lower compared to the PBS group by day 3, but similar on day 7 (Figure 5D). These results suggest that injection of rhIL8 had anti-inflammatory and therapeutic effects in the murine CLI model, although the effects were not persistent.

## Discussion

Interleukin-8 is a chemokine protein that plays a crucial role in the immune system’s response to tissue damage and inflammation.^26^ When tissue is damaged, IL8 released from immune cells recruits neutrophils and monocytes/macrophages to the site of injury. These cells then help to clear away debris and pathogens and release factors that promote tissue repair and regeneration.^27^ Furthermore, IL8 has been reported to have an immune-suppressive polarizing effect on macrophages in various cancers including breast cancer,^14^ bladder cancer,^15^ gastric cancer,^16^ and ovarian cancer.^17^ To our knowledge, immune-modulating potential of IL8 in other diseases has not yet been reported. We conducted a hypothetical verification to investigate whether IL8 secreted from an hASC spheroid (FECS-Ad), which is formed on an FGF2-tethered solid surface, exerts a protective effect against tissue degeneration and immunomodulatory effects that contribute to healing in a critical limb ischemia model. IL8 was shown to be effective in resisting tissue disintegration, as evidenced by data such as conserved limb ratio, histology, immunofluorescence staining of tissue, and microvascular apoptosis. Additionally, IL8 demonstrated an impact on immunomodulation, shifting toward anti-inflammatory phase, as supported by the immunofluorescence staining of M1 and M2 macrophages, flow cytometry analysis, in vivo mRNA expressions of pro- and anti-inflammation markers (Figure S3, see online supplementary material for a color version of this figure), and in vitro indirect co-culture of FECS-Ad and THP-1 cells (Figure S4, see online supplementary material for a color version of this figure). Taken together, our results suggest an interpretation of the roles of IL8 in immune modulation and tissue protection during ischemic injury.

In this study, we demonstrated that IL8 plays a significant role in mediating anti-inflammatory responses. However, numerous studies have also highlighted that IL8’s capacity to induce pro-inflammatory effects. For instance, in experiments involving transgenic mice carrying the human IL8 gene, elevated levels of IL8 were observed in both damaged tissues and blood, leading to increased immune cell migration and promoting carcinogenesis.^28^ Additionally, the injection of an adenovirus engineered to express 7500 ng/mL of IL8 in vitro resulted in ulcer formation in a skin graft model in vivo.^29^ High expression levels of IL8 have been linked to chronic inflammation and various pathologic conditions.^30^^,^^31^ Given that FECS-Ad secretes IL8 at approximately 500 ng/mL, it is likely that the amount of IL8 secreted in our study was lower than that in studies reporting its pro-inflammatory effects. The expression of the IL8 gene is tightly regulated, and the recruitment of immune cells to sites of injury is finely modulated by this regulation.^32^ When a substantial amount of IL8 is administered, there is an increased accumulation immune cells in the bloodstream, which can prolong the inflammatory response.^33^ This suggests that IL8 can exhibit contradictory effects, contributing to either anti-inflammatory or chronic inflammatory ­processes depending on its concentration. Given that the anti-inflammatory properties of IL8 have been documented in various cancer models and were first demonstrated in ischemic injury, further investigation into the relationship between IL8 concentration and its anti-inflammatory effects is warranted. This research could pave the way for the development of innovative cell therapies that leverage IL8 as an anti-inflammatory agent, challenging the prevailing notion that IL8 should solely be suppressed or eliminated in therapeutic contexts.

Next, we inquired the effects of IL8 on endothelial cells, in addition to its immunomodulatory effects. Hindlimb ischemia induces endothelial cell apoptosis in the early stage of injury,^34^ as demonstrated in PBS-injected tissues subjected to HLI (Figure S5A and C, see online supplementary material for a color version of this figure). Transplantation of FECS-Ad, which secretes IL8 protein, improved the microvascular survival (Figure S5A and B, see online supplementary material for a color version of this figure). IL8 has been demonstrated to have antiapoptotic effects on various cell types, including neutrophils,^35^^,^^36^ endothelial cells,^37^^,^^38^ and some cancer cells^21^^,^^39^ by activating pro-survival signaling and regulating the expression of apoptotic proteins. Additionally, it has been reported that infarct size is reduced by IL8 administration in brain ischemic injury.^12^ Therefore, the observation that blood vessels deterioration was more severe in tissue injected with hASC or IL8 knocked-down FECS-Ad compared to those injected with normal FECS-Ad can be attributed to this effect. However, it is important to note that while inflammation can induce endothelial cell apoptosis, these processes are highly complex and context-dependent. In some cases, inflammation may also lead to endothelial cell proliferation and tissue repair.^40^^,^^41^

In addition to its role in promoting endothelial survival, it is plausible that IL8 may exert anti-apoptotic effects on muscle tissue. In ischemic environments induced by exercise, human skeletal muscle cells secrete IL8 as a type of myokine, which exerts its effects locally.^42^ Given that the expression of skeletal muscle C-X-C motif chemokine receptor 2 (CXCR2), a receptor for IL8, increases following concentric exercise,^43^ IL8 may be involved in regulating the survival of skeletal muscle in an autocrine manner under stress conditions such as ischemia. Furthermore, IL8 is crucial for the differentiation of myoblasts into mature muscle cells and promotes the production of myocilin, a protein essential for skeletal muscle growth.^44^^,^^45^ However, there are conflicting reports regarding IL8’s role in muscle atrophy in vitro and in vivo.^46^^,^^47^ The divergent effects of IL8 on muscle hypertrophy and atrophy may be attributed to the timing of administration. When IL8 is administered during the stage when myoblasts are proliferating and transitioning to myotube differentiation, it promotes this differentiation, leading to the formation of larger and longer myotubes.^44^^,^^45^ Conversely, when IL8 is administered after differentiation has already occurred, it causes the myotubes to shrink.^46^^,^^47^ The response of skeletal muscle to cytokines during regeneration is reported to depend on the stage of myogenesis.^48^ IL8 released from FECS-Ad may act on regenerating muscle tissues, promoting the differentiation and hypertrophy of muscle fibers. Further investigation is required to determine whether IL8 secreted from FECS-Ad directly influences muscle cells during ischemic injury. Alternatively, muscle regeneration may have occurred due to the anti-inflammatory effects of IL8. It is well established that muscle stem cells are activated and proliferate to facilitate regeneration immediately after muscle tissue damage. However, in an inflammatory microenvironment, muscle stem cell division is prematurely halted, and differentiation begins, resulting in incomplete regeneration. For this reason, the FECS-Ad injection group likely exhibited superior muscle size and area compared to other groups, as an anti-inflammatory macrophage and environment were established from day 3, thereby promoting enhanced regeneration. However, this study’s in vivo nature presents limitations in exploring the underlying mechanisms. Consequently, it remains uncertain whether the observed tissue-protective effects and enhanced myoregeneration are attributable to the interaction between IL8 and muscle tissue or are primarily a result of IL8’s anti-inflammatory properties.

After confirming the anti-inflammatory and tissue-protective effects of FECS-Ad, which secretes IL8, we administered IL8 protein alone intramuscularly to the CLI model. Although the therapeutic effect of necrotic damage in hindlimb in the rhIL8-injected group was not comparable to that in the FECS-Ad-injected group, limb loss did not occur in the rhIL8 1×, 2× injected group (Figure 5B). The limited therapeutic effect may be attributed to the short-term retention of injected rhIL8 in the tissue, as shown in Figure S6B (see online supplementary material for a color version of this figure). It was observed that the anti-inflammatory effects of rhIL8 were not sustained and the condition reverted to a pro-inflammatory state (Figure 5B and C). In contrast, FECS-Ad releases a variety of paracrine factors, such as PDGF, HGF, and FGF2, in addition to IL8.^9^^,^^49^ As a result, FECS-Ad may have a superior therapeutic and anti-inflammatory effect compared to the administration of IL8 protein. In conditions with complex pathological phenomena, such as critical limb ischemia, which involves inflammatory responses, apoptosis, muscle regeneration, and tissue remodeling, stem cell therapy may provide more effective treatment than cytokine or growth factor injections. This is due to its potential for comprehensive treatment through the sustained secretion of various factors.^50–52^ Whereas IL8 injection offers a transient, one-shot effect, FECS-Ad, as a stem cell therapy, actively participates in the healing process by interacting with the surrounding environment and sustaining both anti-inflammatory and regenerative effects. In summary, increased secretion of IL8 from FECS-Ad serves as both a potential protector against tissue degeneration and an immune suppressor. IL8 from FECS-Ad not only reduces the number of apoptotic cells but also mitigates immune response deterioration by directing the macrophages toward an anti-inflammatory M2 phenotype. Our current understanding of IL8’s role in immune modulation in the CLI model is extremely limited. Further studies are needed to elucidate IL8’s involvement in immune suppression, including its effects on T cells, B cells, NK cells, and other immune cells.

Aside from the interactions between IL-8 and immune cells, stem cells both influence and are influenced by the physiological environment into which they are introduced. Consequently, stem cell therapies that show efficacy in animal models may not achieve the expected therapeutic outcomes in humans.^53^ However, in this study, we preconditioned the stem cells via FGF2 priming to establish a defined response profile, and therefore, we anticipate that the anti-inflammatory effects will also be observed in human applications.^54^ Nevertheless, considering that the persistence of stem cells in the human body may differ from that in mouse models, which have a faster metabolic rate, further investigations are warranted to optimize the dosage and administration schedule for the clinical translation of FECS-Ad. Expanding this knowledge may deepen our understanding of human immunology and improve the therapeutic application of FECS-Ad in ischemic diseases.

## Supplementary Material

szaf051_Supplementary_Data

## Data Availability

The data that support the findings of this study are available from the corresponding author upon reasonable request. Extended Materials and Methods are provided in the Supplementary Material, along with detailed information about the resources used in this study.
