# Supplementary material for: Fibroblast Growth Factor 2-engaged cell spheroid for stem cell therapy: role of Interleukin 8 in the immune-modulatory effectiveness in the critical limb ischemia model
Source: Stem Cells Transl Med. 2025 Nov 14;14(11):szaf051. doi: 10.1093/stcltm/szaf051 (PMC12616473; doi:10.1093/stcltm/szaf051)
Supplement: szaf051_Supplementary_Data [file szaf051_supplementary_data.zip › SCTM-25-0013.R1 SUPPLEMENTAL INFORMATION.docx]

**SUPPLEMENTAL MATERIALS**

**FGF2-engaged cell spheroid for stem cell therapy: Role of IL8 in the immune-modulatory effectiveness in the critical limb ischemia model.**

Eunyeong Kim^1,2^, Yunji Joo^1,2^, Jong-W.an Kim^3^, Sang-Heon Kim^1,2,^*

^1^Center for Biomaterials, Biomedical Research Institute, Korea Institute of Science and Technology (KIST), Seoul 02792, Republic of Korea

^2^Division of Bio-Medical Science and Technology, KIST School, Korea University of Science and Technology, Seoul 02792, Republic of Korea

^3^S.Biomedics Co., Ltd., Seoul 04797, Republic of Korea

| **Sample** | **Count** | **cells** | **cells/F480+** | **cells/F480+/CD86+** | **cells/F480+/CD206+** |
| --- | --- | --- | --- | --- | --- |
| Normal leg_F480+CD86+ (1) | 10000 | 9595 | 98 | 10 |  |
| Normal leg_F480+CD206+ (1) | 10000 | 9651 | 92 |  | 33 |
| Normal leg_F480+CD86+ (2) | 10000 | 9527 | 189 | 29 |  |
| Normal leg_F480+CD206+ (2) | 10000 | 9554 | 146 |  | 53 |
| Normal leg_F480+CD86+ (3) | 10000 | 9663 | 97 | 11 |  |
| Normal leg_F480+CD206+ (3) | 10000 | 9614 | 80 |  | 37 |
| D1_F480+CD86+ (1).fcs | 10000 | 9774 | 157 | 12 |  |
| D1_F480+CD206+ (1).fcs | 10000 | 9793 | 110 |  | 24 |
| D1_F480+CD86+ (2).fcs | 10000 | 9729 | 160 | 14 |  |
| D1_F480+CD206+ (2).fcs | 10000 | 9569 | 128 |  | 30 |
| D1_F480+CD86+ (3).fcs | 10000 | 9735 | 179 | 16 |  |
| D1_F480+CD206+ (3).fcs | 10000 | 9735 | 256 |  | 44 |
| D3_PBS_F480+CD86+ (1).fcs | 10000 | 9660 | 277 | 63 |  |
| D3_PBS_F480+CD206+ (1).fcs | 10000 | 9662 | 274 |  | 122 |
| D3_PBS_F480+CD86+ (2).fcs | 10000 | 9654 | 200 | 54 |  |
| D3_PBS_F480+CD206+ (2).fcs | 10000 | 9595 | 225 |  | 96 |
| D3_PBS_F480+CD86+ (3).fcs | 10000 | 9501 | 149 | 39 |  |
| D3_PBS_F480+CD206+ (3).fcs | 10000 | 9458 | 163 |  | 83 |
| D3_hASC_F480+CD86+ (1).fcs | 10000 | 9654 | 289 | 79 |  |
| D3_hASC_F480+CD206+ (1).fcs | 10000 | 9724 | 330 |  | 175 |
| D3_hASC_F480+CD86+ (2).fcs | 10000 | 9674 | 247 | 70 |  |
| D3_hASC_F480+CD206+ (2).fcs | 10000 | 9701 | 323 |  | 189 |
| D3_hASC_F480+CD86+ (3).fcs | 10000 | 9712 | 280 | 83 |  |
| D3_hASC_F480+CD206+ (3).fcs | 10000 | 9641 | 310 |  | 185 |
| D3_FECS-Ad_F480+CD86+ (1).fcs | 10000 | 9387 | 651 | 93 |  |
| D3_FECS-Ad_F480+CD206+ (1).fcs | 10000 | 9460 | 472 |  | 248 |
| D3_FECS-Ad_F480+CD86+ (2).fcs | 10000 | 9463 | 554 | 95 |  |
| D3_FECS-Ad_F480+CD206+ (2).fcs | 10000 | 9457 | 441 |  | 205 |
| D3_FECS-Ad_F480+CD86+ (3).fcs | 10000 | 9384 | 613 | 95 |  |
| D3_FECS-Ad_F480+CD206+ (3).fcs | 10000 | 9391 | 478 |  | 237 |
| D3_IL8KD FECS-Ad_F480+CD86+ (1).fcs | 10000 | 9501 | 147 | 45 |  |
| D3_IL8KD FECS-Ad_F480+CD206+ (1).fcs | 10000 | 9599 | 157 |  | 80 |
| D3_IL8KD FECS-Ad_F480+CD86+ (2).fcs | 10000 | 9581 | 151 | 37 |  |
| D3_IL8KD FECS-Ad_F480+CD206+ (2).fcs | 10000 | 9529 | 138 |  | 75 |
| D3_IL8KD FECS-Ad_F480+CD86+ (3).fcs | 10000 | 9603 | 118 | 31 |  |
| D3_IL8KD FECS-Ad_F480+CD206+ (3).fcs | 10000 | 9603 | 106 |  | 63 |
| D7_PBS_F480+CD86+ (1).fcs | 10000 | 9596 | 1145 | 423 |  |
| D7_PBS_F480+CD206+ (1).fcs | 10000 | 9631 | 1027 |  | 604 |
| D7_PBS_F480+CD86+ (2).fcs | 10000 | 9572 | 1173 | 446 |  |
| D7_PBS_F480+CD206+ (2).fcs | 10000 | 9625 | 992 |  | 609 |
| D7_PBS_F480+CD86+ (3).fcs | 10000 | 9555 | 1163 | 441 |  |
| D7_PBS_F480+CD206+ (3).fcs | 10000 | 9551 | 1032 |  | 595 |
| D7_ASC_F480+CD86+ (1).fcs | 10000 | 9578 | 933 | 141 |  |
| D7_ASC_F480+CD206+ (1).fcs | 10000 | 9552 | 897 |  | 368 |
| D7_ASC_F480+CD86+ (2).fcs | 10000 | 9613 | 872 | 110 |  |
| D7_ASC_F480+CD206+ (2).fcs | 10000 | 9550 | 828 |  | 308 |
| D7_ASC_F480+CD86+ (3).fcs | 10000 | 9556 | 731 | 115 |  |
| D7_ASC_F480+CD206+ (3).fcs | 10000 | 9552 | 897 |  | 368 |
| D7_FECS-Ad_F480+CD86+ (1).fcs | 10000 | 9725 | 308 | 79 |  |
| D7_FECS-Ad_F480+CD206+ (1).fcs | 10000 | 9603 | 335 |  | 180 |
| D7_FECS-Ad_F480+CD86+ (2).fcs | 10000 | 9689 | 242 | 67 |  |
| D7_FECS-Ad_F480+CD206+ (2).fcs | 10000 | 9631 | 332 |  | 201 |
| D7_FECS-Ad_F480+CD86+ (3).fcs | 10000 | 9626 | 257 | 63 |  |
| D7_FECS-Ad_F480+CD206+ (3).fcs | 10000 | 9734 | 284 |  | 166 |
| D7_IL8KD FECS-Ad_F480+CD86+ (1).fcs | 10000 | 9627 | 927 | 235 |  |
| D7_IL8KD FECS-Ad_F480+CD206+ (1).fcs | 10000 | 9602 | 945 |  | 522 |
| D7_IL8KD FECS-Ad_F480+CD86+ (2).fcs | 10000 | 9646 | 913 | 235 |  |
| D7_IL8KD FECS-Ad_F480+CD206+ (2).fcs | 10000 | 9603 | 863 |  | 466 |
| D7_IL8KD FECS-Ad_F480+CD86+ (3).fcs | 10000 | 9610 | 652 | 205 |  |
| D7_IL8KD FECS-Ad_F480+CD206+ (3).fcs | 10000 | 9636 | 706 |  | 410 |

**Table S1. Raw flow cytometry data for macrophage polarization in ischemic tissue.**
Cell numbers in each group were quantified after gating for F4/80+ and CD86+ or CD206+ cells in the medial thigh of mice injected with PBS, hASC, FECS-Ad, or IL8KD FECS-Ad following hindlimb ischemia surgery. D1 denotes Day1, D3 denotes Day3, and D7 denotes Day7.

**A**

| **Target antigen** | **Vendor or Source** | **Catalog #** | **Working concentration** |
| --- | --- | --- | --- |
| Laminin | Sigma Aldrich | L9393 | IF (1:200) |
| iNOS  (M1 mcrophage) | Millipore | 06-573 | IF (1:200) |
| CD206  (M2 macrophage) | Abcam | Ab64693 | IF (1:200) |
| F4/80  (pan-macrophage) | BD Pharmingen | BD-565409 | IF (1:50) |
| Caspase 8 | SantaCruz Biotechnolgy | Sc-5263 | IF (1:200) |
| Cleaved caspase 3 | Cell Signaling | 9579 | IF (1:200) |
| eMHC | DSHB | F1.652 | IF (1:30) |
| CD31 | Thermo Fisher | MA1-26196 | IF (1:50) |
| HNA | Abcam | ab191181 | IF (1:200) |
| IL8 | Cell Signaling | 94407 | IF (1:100) |
| F4/80 | Biolegend | 123116 | FC (1:200) |
| CD206 | Biolegend | 141706 | FC (1:200) |
| CD86 | Biolegend | 105006 | FC (1:200) |

**B**

| **Primers** | **Forward sequence (5’-3’)** | **Reverse sequence (5’-3’)** |
| --- | --- | --- |
| miNOS | GAGACAGGGAAGTCTGAAGCA | CCAGCAGTAGTTGCTCCTCTT |
| mTNFα | GCCACCACGCTCTTCTGTCT | GTCTGGGCCATGGAACTGAT |
| mCD206 | CAGGTGTGGGCTCAGGTAGT | TGTGGTGAGCTGAAAGGTGA |
| mTGFβ | CTCCCGTGGCTTCTAGTGC | GCCTTAGTTTGGACAGGATCTG |
| mGapdh | CATCACTGCCACCCAGAAGACTG | ATGCCAGTGAGCTTCCCGTTCAG |
| hCxcl10 | GGTGAGAAGAGATGTCTGAATCC | GTCCATCCTTGGAAGCACTGCA |
| hCcl1 | TGTGCCTCTGAACCCATCCAAC | ACCAGCTCCATCTGCTCCAATG |
| hCD209 | TGCTGAGGAGCAGAACTTCC | GTTGGGCTCTCCTCTGTTCC |
| hIL4 | CCTCACAGAGCAGAAGAACAC | TGTCGAGCCGTTTCAGGAAT |
| hVEGF | TTGCCTTGCTGCTCTACCTCCA | GATGGCAGTAGCTGCGCTGATA |
| hGAPDH | GTCTCCTCTGACTTCAACAGCG | ACCACCCTGTTGCTGTAGCCAA |
| hIL8 | GAGAGTGATTGAGAGTGGACCAC | CACAACCCTCTGCACCCAGTTT |

m; mouse, h; human

**Table S2. Antibodies and primers used in this study**

(A) Antibodies used for immunofluorescence. (B) Nucleotide primer sequence used for RT-qPCR analysis.

**
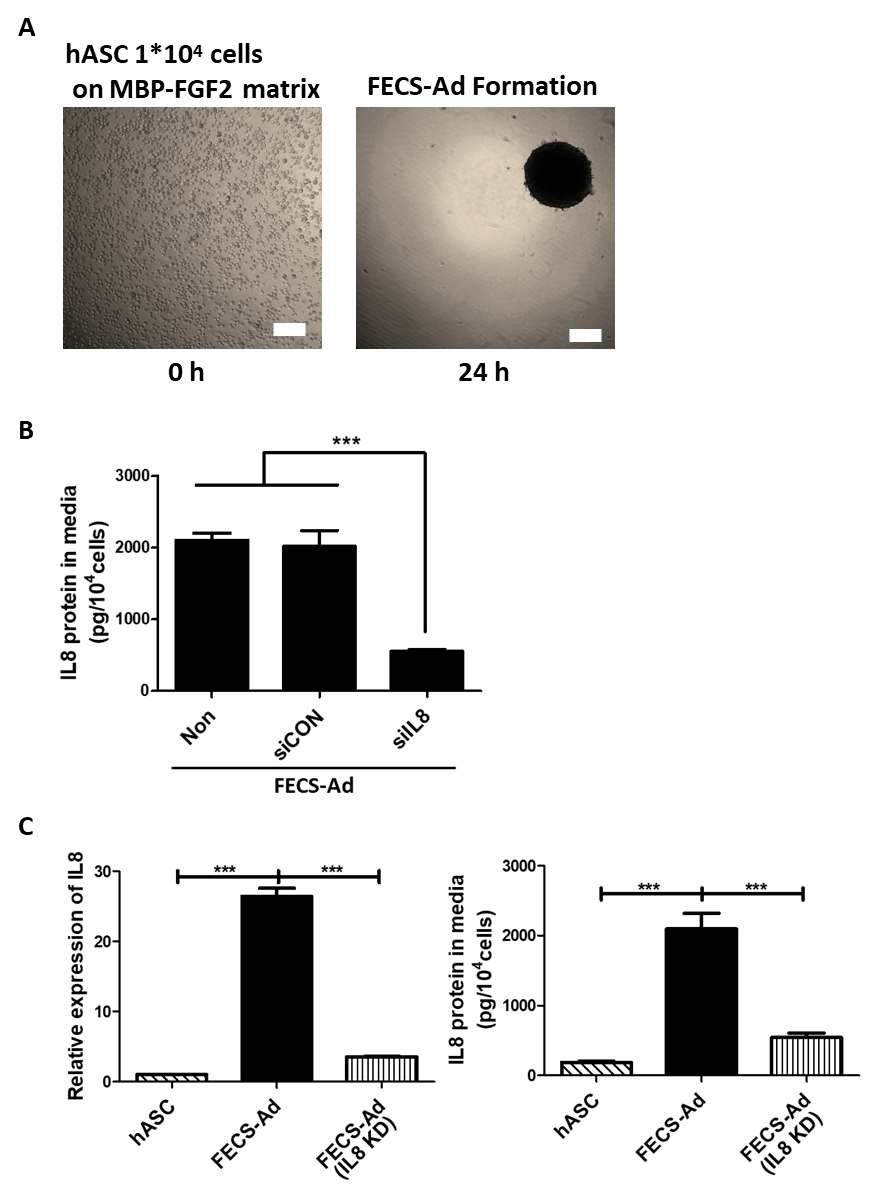
**

**Figure S1. Formation of FECS-Ad and IL8 expression in FECS-Ad.** **(A)** Formation of FECS-Ad by seeding 1 × 10^4^ hASC onto an FGF2-tethered 384-well plate for 24 hours. Scale bar = 200 μm **(B)** IL8 secretion from FECS-Ad and its knockdown achieved through transfection with IL8 siRNA (siIL8) and siRNA negative control (siCON). in FECS-Ad using **(C)** Comparison of IL8 gene and protein expression in FECS-Ad, hASC, and IL8 KD FECS-Ad.


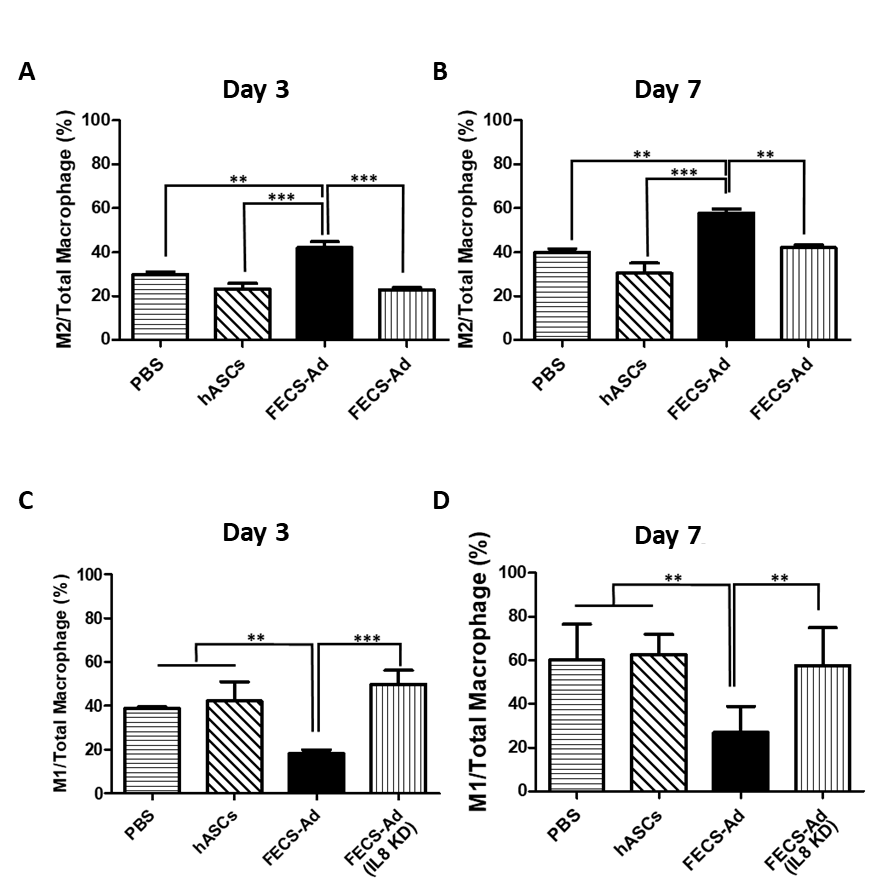


**Figure S2. Quantification of M1 or M2 marker positive area relative to total macrophage area.** Quantification bar graphs show the positive area for M1 or M2 marker per total macrophage area at days 3 and 7. Representative images are presented in Figures 2A and 2B. n = 5 per group. Data are expressed as mean ± SEM. Statistical significance was determined using one-way ANOVA with Tukey’s test (**p < 0.01, ***p < 0.001).


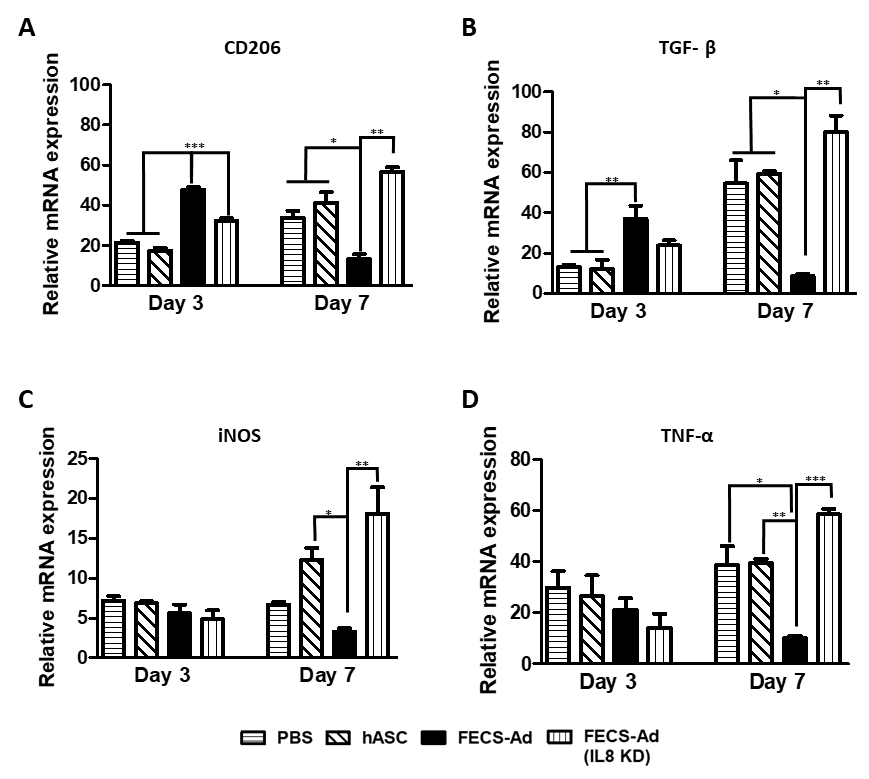


**Figure S3. Anti-inflammatory polarization of macrophages by IL8 from FECS-Ad in ischemia-induced thigh muscle at days 3 and 7.** RT-qPCR data showing the expression levels of anti- inflammatory markers **(A, B)** and pro-inflammatory markers **(C, D)** in the medial thighs of mice following hindlimb ischemia and inoculation of PBS, hASC, FECS-Ad, or IL8 silenced FECS-Ad, relative to the normal leg control. n=3 per group. Data are normalized to GAPDH and presented as mean ± SEM. Statistical significance was assessed using one-way ANOVA followed by Tukey’s test (*p < 0.05, **p < 0.01, ***p < 0.001).


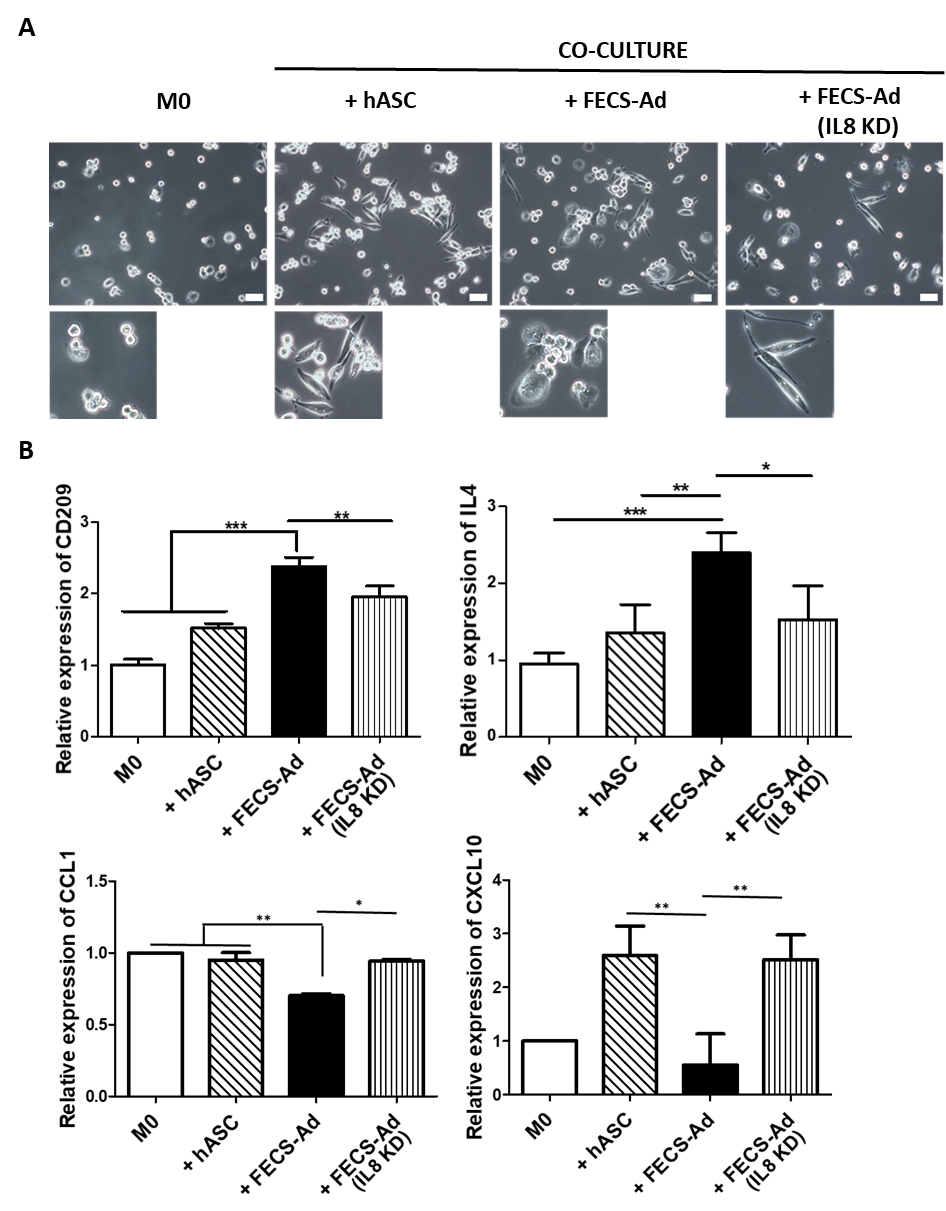


**Figure S4. Anti-inflammatory effects of IL8 secreted from FECS-Ad on M0 THP-1 human macrophages (M0) *in vitro*.** **(A)** Morphological phenotype of M0 THP-1 macrophage after two days of indirect co-culture with hASC, FECS-Ad, or IL8 KD FECS-Ad using a trans-well system. Scale bar = 50 μm. **(B)** RT-qPCR expression data of the co-cultured M0 THP-l cells relative to conventionally activated M0 THP-1 cells (n = 3). CD209, and IL4 were used as an anti-inflammatory markers, while CXCL10 and CCL1 were served as pro-inflammatory markers.


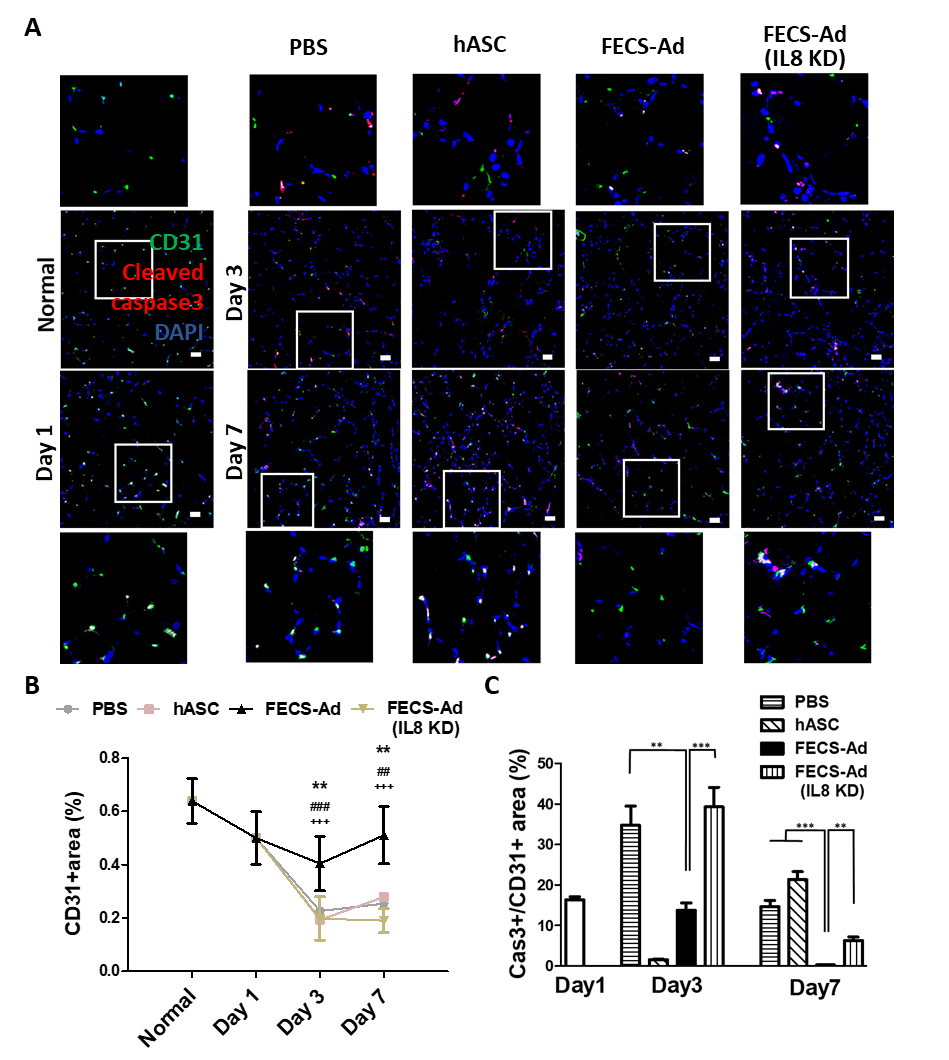


**Figure S5. Roles of IL8 in the microvascular protection of FECS-Ad in mouse hindlimb ischemia.** **(A)** Immunofluorescent images of ischemic thigh muscle stained for CD31 and cleaved caspase 3, collected from mice treated with PBS, hASC, FECS-Ad, or IL8 KD FECS-Ad at days 3 and 7. Nuclei were counterstained with DAPI. Scale bar = 20 μm. **(B)** Quantification of CD31-positive area in ischemic thigh tissue over 7 days. n = 5 per group. Statistical analysis was performed one-way ANOVA with Tukey’s test (**p < 0.01 against the PBS group. ##p < 0.01, ###p < 0.001 against the hASC group, +++p < 0.001 against IL8 KD FECS-Ad group). **(C)** Quantification of cleaved caspase3-positive (Cas3+) cells relative to total CD31-positive cells. All data are presented as mean ± SEM. n = 5 per group. Statistical significance was determined using one-way ANOVA with Tukey’s test (**p < 0.01, ***p < 0.001).

**Figure S6. IL8 secretion by FECS-Ad in ischemic thigh muscle. (A)** Immunostaining for HNA and IL8 was conducted following the injection of PBS, hASC, FECS-Ad, and IL8 KD FECS-Ad after hindlimb ischemia induction in a murine model at days 3 and 7. **(B)** No detectable IL8 remained in the ischemia-induced thigh tissue of mice after the injection of rhIL8. Nuclei were counterstained with DAPI. Scale bar = 20 μm.
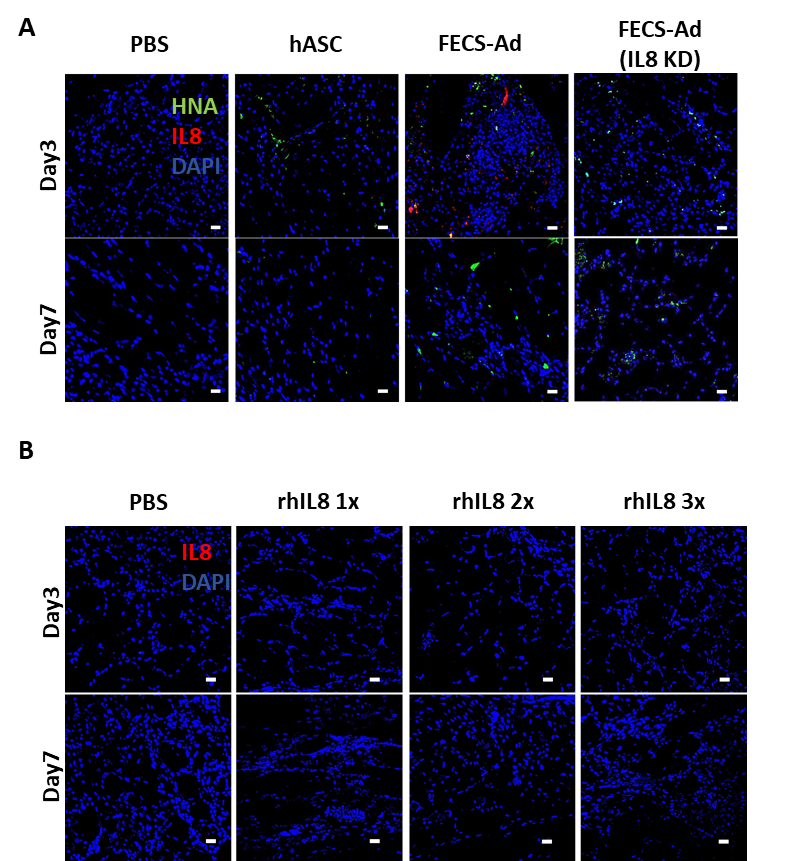


**Supplemental Methods**

- Enzyme-linked immunosorbent assay (ELISA)
Conditioned media were prepared, and the protein level of IL8 was quantified using an ELISA kit (R&D Systems, DY208) according to the manufacturer’s instructions. The optical density was measured at 450 nm and 540 nm using a GloMax Discover Microplate Reader (Promega). The readings at 540 nm were subtracted from those at 450 nm for analysis.

- Reverse transcription and polymerase chain reaction (PCR)
For RNA extraction from cells, RNeasy mini kit (Qiagen; 74104) was used following the manufacturer’s protocol. RNA concentration was quantified using a NanoDrop spectrophotometer (Thermo Fisher Scientific; ND1000). cDNA synthesis was performed using 1 μg of RNA with the Superscript VILO cDNA synthesis kit (Invitrogen; 100011931) according to the manufacturer’s instructions. For RNA extraction from tissues, samples were homogenized in 1 mL TRIzol Reagent (Life Technologies; 15596-018) and vortexed after the addition of 100 μL chloroform (VWR, 0757) followed by a 5-minute incubation at room temperature. The solution was centrifuged at 12000 X g for 15 minutes at 4^◦^C. The upper aqueous layer was transferred to a new tube and 250 μL of isopropanol (Merck KGaA; 109634) was added, followed by 10 minutes incubation at room temperature. The solutions are centrifuged at 12000 X g for 10 minutes at 4^◦^C, and the resulting pellet was resuspended in 70% ethanol. The ethanol-washed solution was centrifuged at 7500 X g for 5 minutes at 4^◦^C, and the upper solution was removed. The remaining pellet was dried for 10 minutes and resuspended in 30 μL of DNAse/RNAse-Free DEPC-Treated Water (Invitrogen; 46-2224). cDNA synthesis was performed as described above for subsequent RT-qPCR. Gene expression levels were determined using SYBR Premix Ex Taq (Takara; RR420) and quantitative real-time PCR with the ABI 7500 Real-Time System (Applied Biosystems). All primer sequence information is provided in the Major Resources Table.
